# Thickness-Dependent Interface Polarity in Infinite-Layer Nickelate Superlattices

**DOI:** 10.1021/acs.nanolett.3c00192

**Published:** 2023-04-07

**Authors:** Chao Yang, Roberto A. Ortiz, Yi Wang, Wilfried Sigle, Hongguang Wang, Eva Benckiser, Bernhard Keimer, Peter A. van Aken

**Affiliations:** † Max Planck Institute for Solid State Research, Stuttgart 70569, Germany; ‡ Center for Microscopy and Analysis, Nanjing University of Aeronautics and Astronautics, Nanjing 210016, P. R. China

**Keywords:** infinite-layer nickelates, polar interface, oxygen distribution, 4D-STEM, EELS, superlattice

## Abstract

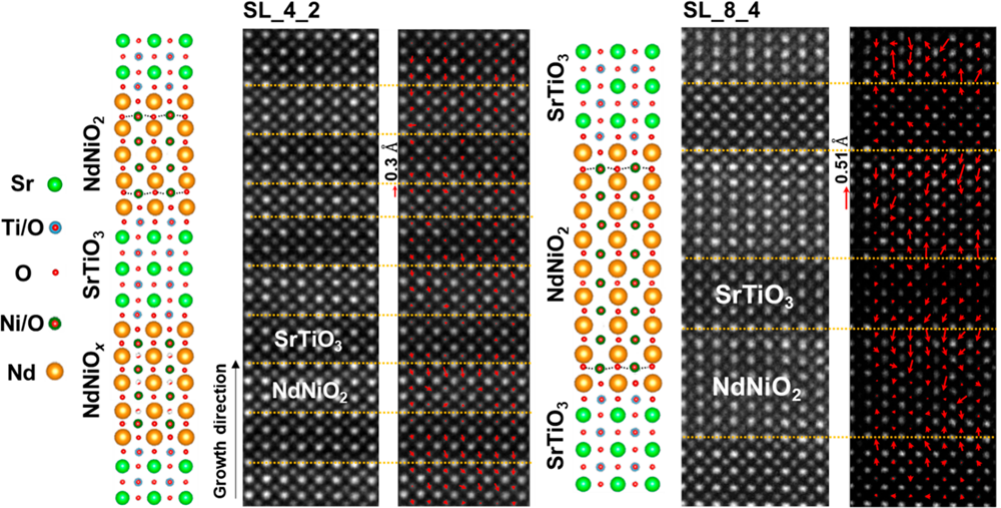

The interface polarity plays a vital role in the physical properties of oxide heterointerfaces because it can cause specific modifications of the electronic and atomic structure. Reconstruction due to the strong polarity of the NdNiO_2_/SrTiO_3_ interface in recently discovered superconducting nickelate films may play an important role, as no superconductivity has been observed in the bulk. By employing four-dimensional scanning transmission electron microscopy and electron energy-loss spectroscopy, we studied effects of oxygen distribution, polyhedral distortion, elemental intermixing, and dimensionality in NdNiO_2_/SrTiO_3_ superlattices grown on SrTiO_3_ (001) substrates. Oxygen distribution maps show a gradual variation of the oxygen content in the nickelate layer. Remarkably, we demonstrate thickness-dependent interface reconstruction due to a polar discontinuity. An average cation displacement of ∼0.025 nm at interfaces in 8NdNiO_2_/4SrTiO_3_ superlattices is twice larger than that in 4NdNiO_2_/2SrTiO_3_ superlattices. Our results provide insights into the understanding of reconstructions at NdNiO_2_/SrTiO_3_ polar interfaces.

Complex oxide superlattices provide a powerful platform for tuning the interactions between charge, spin, orbital, and lattice degrees of freedom. By reducing the individual layer thicknesses to a few atomic layers and successively repeating them in the superlattice structure, interface reconstructions can dominate the macroscopic properties.^1−3^ An effective approach to modify the interface properties in complex oxide heterostructures is via adjustment of the oxygen octahedral structure, charge transfer, electronic confinement, magnetic exchange interactions, and so on. For example, thickness-dependent oxygen octahedral rotations in the NdNiO_3_/SrTiO_3_ superlattice significantly affect the metal–insulator transition and antiferromagnetic transition.^4^ That is also strongly affected by oxygen stoichiometry. The polar discontinuity at the NdNiO_3_/SrTiO_3_ interface may result in interface reconstruction, inducing the formation of oxygen vacancies. Besides the reconstructions mentioned above, layer-selective topotactic reduction is another very interesting way to change the properties. In the topotactic reduction process^5−8^ the formal electronic configuration of Ni changes from 3d^7^ to 3d^9^ by successive apical oxygen removal. Unconventional superconductivity has been first observed in a infinite-layer Sr-doped NdNiO_2_ film^9^ and was later reproduced in A_0.8_B_0.2_NiO_2_ (A: La, Nd, Pr; B: Ca)^9−11^ and a Nd_6_Ni_5_O_12_ film;^12^ however, to date no superconductivity is found in bulk samples.^13^ Some studies have investigated the role of the interface properties and the film geometry for superconductivity. For example, theoretical calculations have predicted the formation of a two-dimensional electron gas as a result of an interface reconstruction at the polar NdNiO_2_/SrTiO_3_ interface,^14,15^ similar to the polar LaAlO_3_/SrTiO_3_ interface.^16−20^ In principle, an abrupt interface between [Nd]^3+^ and [TiO_2_]^0^ layers can induce a polar discontinuity, leading to a built-in electrostatic field that would result in a polar catastrophe. However, the occurrence of electronic and/or atomic reconstruction can avoid such a polar instability. He et al.^21^ verified by DFT calculations that an atomic reconstruction is more energetically favorable than an electronic reconstruction and predicted an interface configuration with residual apical oxygen atoms as well as Ni displacements. A single intermediate Nd(Ti,Ni)O_3_ layer was observed by atomic-resolution EELS in a NdNiO_2_ single film grown on a (001)-oriented SrTiO_3_ single crystal, indicating that atomic reconstruction at the polar interface mitigates the polar instability.^22^ In addition, superlattice structures were proposed to introduce hole doping in their infinite-layer stacks through interface engineering,^23^ providing the possibility of achieving superconductivity in nickelates, without disorder introduced by alkaline-earth doping. This so-called superlattice approach relies on the possibility to tune the doping level through the infinite-layer stack thickness. While a first realization with LaNiO_2+*x*_/LaGaO_3_ interfaces turned out to be not superconducting as holes get trapped at the interface,^23^ different atomic or electronic reconstruction at NdNiO_2_/SrTiO_3_ interfaces offer different possibilities to tune the properties of superlattices. To this end, a detailed experimental investigation of the oxygen octahedra and the stoichiometry at the interfaces of the superlattice with atomic precision is essential.

In this work, NdNiO_2_/SrTiO_3_ superlattices with different stacking thicknesses were synthesized in two steps: (i) growing the perovskite phase of NdNiO_3_/SrTiO_3_ superlattices on a (001)-oriented SrTiO_3_ substrate by pulsed-laser deposition (PLD) and (ii) reducing samples by soft-chemistry topotactic reduction. By employing four-dimensional scanning transmission electron microscopy (4D-STEM) and atomically resolved electron energy-loss spectroscopy (EELS), we provide a detailed characterization of the oxygen structure and concentration distribution as well as the electronic structure in NdNiO_2_ (NNO)/SrTiO_3_ (STO) superlattices. We directly image the thickness-related variation of the oxygen concentration, revealing the reduction procedure in the NNO layer. Moreover, we discuss the spacial variation of atomic and local electronic structures across the interfaces in NNO/STO superlattices. We found a gradual variation of the oxygen content from the interface to the inner part of the nickelate layer stack. Our results provide a picture of the spacial extend of the reconstructions related to the polar interfaces, which is instructive for understanding the thickness-dependent properties of infinite-layer superlattices.

In Figure 1, atomically resolved STEM-EELS chemical mapping identifies the chemical components and distributions across all interfaces in an 8 unit cell thick NNO/4 unit cell thick STO superlattice (SL_8_4). The high-angle annular dark-field (HAADF) image in Figure 1a gives the atomic structure of the NNO and the STO layers. The corresponding elemental maps of Sr-L_2, 3_ (b), Ti-L_2, 3_ (c), Nd-M_4, 5_ (d), and Ni-L_2, 3_ (e) edges as well as an RGB overlay (f) are shown on the right side. Figure 1g shows the enlarged HAADF image from the region marked by a white dashed box in Figure 1a, and the accordingly normalized intensity profiles of (h) Ni and Ti and (i) Nd and Sr reveal the apparent elemental interdiffusion at the interfaces. The interdiffusion lengths of Ni/Ti and Nd/Sr amount to 1–2 unit cells on both sides of the interface. The cation intermixing is not homogeneous at different interfaces. For example, the Ni/Ti ratio is ∼3 (Nd/Sr ratio of ∼3) at interface A and ∼1 (Nd/Sr ratio of ∼0.95) at interface B in the NNO layer, indicating the instability of the interface structure. Nevertheless, the dominant interface configuration consists of a connection of a TiO_2_-terminated STO surface and a NdO_*x*_*-*terminated NNO surface, which is different from the reported single intermediate Nd(Ti, Ni)O_3_ layer in the NdNiO_2_–SrTiO_3_ (substrate) interface, where the B-site cation is predominantly Ti with some Ni occupancy. The former interface configuration of TiO_2_–NdO_*x*_–NiO_2_–Nd is more polar. Similar interdiffusion lengths of cations and interface configurations occur in the short-periodic 4NNO/2STO superlattice (SL_4_2), as shown in Figure S1. This leads to the presence of Ni and Ti throughout the whole film and of Nd in all STO stacking layers. In comparison, the interface between the substrate and the first NNO layer shows much less intermixing, which shows that the elemental intermixing forms during the growth of the perovskite phase. The cation intermixing is not affected by the chemical reduction procedure, which would mainly affect the oxygen ions. As the reduction energy of Ti–O is higher than that of Ni–O,^14,24,25^ the intermixing of Ti into Ni sites can promote oxygen intercalation in the NNO layer near the interfaces. A theoretical calculation predicted that the formation of residual oxygen in the first NdO layer can effectively avoid the polar instability resulting from the formation of a built-in electrostatic field at the polar interface.^21^

**Figure 1:**
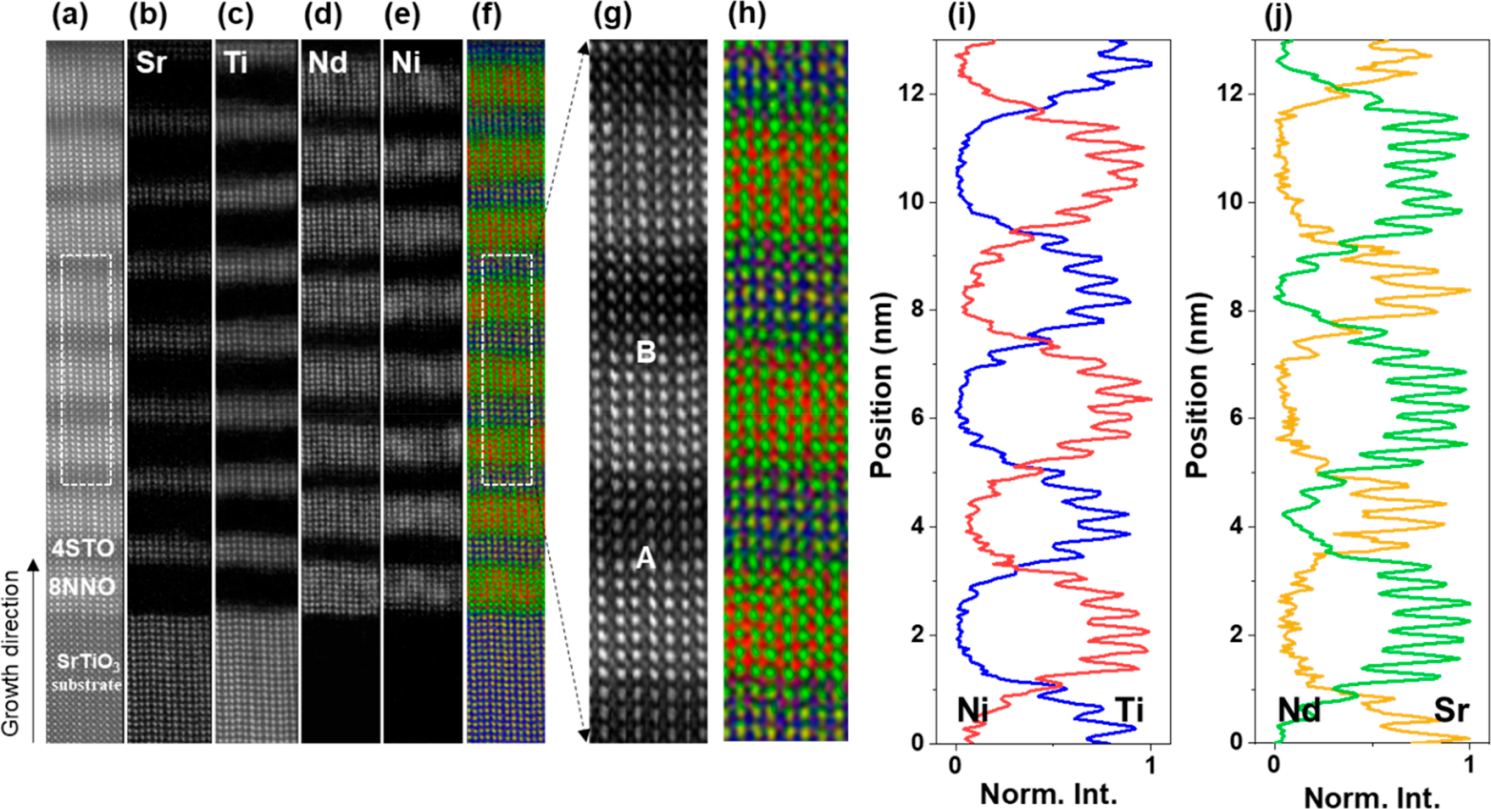
Elemental distribution across the interfaces of an 8NdNiO_2_/4SrTiO_3_ superlattice. (a) An HAADF image for the acquisition of the EELS mapping throughout the film. EELS maps of (b) Sr L_2,3_, (c) Ti L_2,3_, (d) Nd M_4,5_, and (e) Ni L_2,3_ integral white-line intensities. (f) Color-coded map of Sr (yellow), Ti (blue), Nd (green), and Ni (red). (g) An enlarged HAADF image from the regions marked with a white dashed box in (a) and (f), respectively. (h) Color-coded map of Sr (yellow), Ti (blue), Nd (green), and Ni (red) and the corresponding normalized intensity line profiles of (i) Ni and Ti and (j) Nd and Sr. Interfaces A and B marked in (g) show different degrees of elemental intermixing as revealed in (i) and (j).

To investigate the atomic-scale structure of the interfaces, we applied the latest advanced 4D-STEM technique to acquire the oxygen sublattice and distribution at the interfaces, which gives a clear oxygen phase-contrast image.^26^ In Figure 2, we compare the atomic structure of the interfaces in the superlattices SL_4_2 and SL_8_4. Figures 2a and 2g are the sketches of the SL_4_2 and SL_8_4 interface structures, respectively. It is well accepted that the apical oxygen in {NiO_6_} octahedra can be deintercalated more easily than the basal oxygen during a topotactic reduction procedure due to its lower Ni–O bonding energy.^7^ This has also been proven by TEM experiments.^11,12,27^ STO and NNO layers can be identified in the HAADF images for SL_4_2 (Figure 2b) and SL_8_4 (Figure 2h) due to their distinctly different mean inner potentials. As displayed in the integrated center of mass (iCoM) images of SL_4_2 (Figure 2c) and of SL_8_4 (Figure 2j), the apical oxygen positions in the NNO infinite layer have an obviously reduced contrast compared to those in the basal oxygen columns. Hence, the apical oxygen is partially deintercalated in the NNO layer. Compared with the NNO layer, the basal and apical oxygen columns in the STO layer maintain the same contrasts.

**Figure 2:**
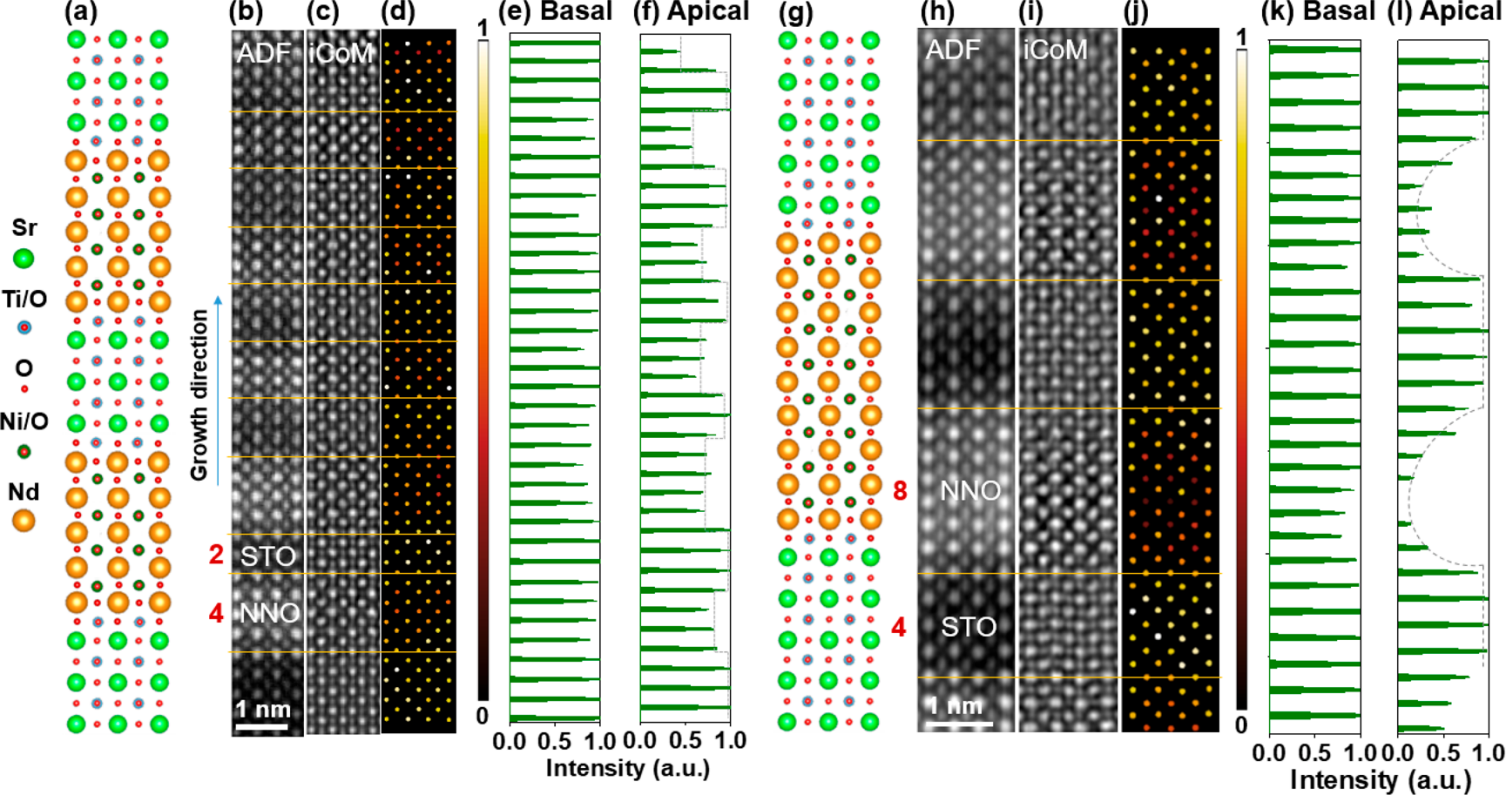
Oxygen distribution and occupancy in the 4NdNiO_2_/2SrTiO_3_ (a–f) and 8NdNiO_2_/4SrTiO_3_ (g–l) superlattices. (a) A schematic diagram of the atomic structure of the 4NdNiO_2_/2SrTiO_3_ superlattice (b) ADF image and (c) iCoM image reconstructed from a 4D-STEM data set for a 4NdNiO_2_/2SrTiO_3_ superlattice. (d) Oxygen intensity map acquired from all oxygen columns in the iCoM image in (c) by Gaussian fitting. The corresponding integrated intensity profiles of (e) basal oxygen and (f) apical oxygen positions. (g) Schematic diagram of the atomic structure of the 8NdNiO_2_/4SrTiO_3_ superlattice. (h) ADF image and (i) iCoM image reconstructed from a 4D-STEM data set for an 8NdNiO_2_/4SrTiO_3_ superlattice. (j) Oxygen intensity map acquired from the iCoM image in (i). The corresponding integrated intensity profiles of (k) basal oxygen and (l) apical oxygen positions.

To understand the deintercalation process of oxygen ions and the variation of the oxygen concentration in the NNO layer, we determined all oxygen positions by Gaussian fitting and then extracted the oxygen intensity map as displayed in Figure 2d for SL_4_2 and in Figure 2j for SL_8_4, where the red color denotes a reduced oxygen contrast. The corresponding integrated intensity profiles of the apical and basal oxygen are presented in Figures 2e,f (SL_4_2) and Figures 2k,l (SL_8_4), respectively. Oxygen intensity maps are normalized to the oxygen intensity in the STO substrate. It is worth mentioning that the intensity of the apical oxygen in the NNO layer for SL_4_2 (Figure 2f) gradually decreases from ∼90% near the STO substrate to ∼30% near the surface, reflecting that the chemical reduction procedure occurs from the surface to the substrate. The locally formed infinite-layer structure near the surface region in the nickelate layer indicates the formation of at most two unit cells of a NNO layer in the SL_4_2 (Figure S2). The intensity profile of the apical oxygen for SL_8_4 (Figure 2l) shows the variation of oxygen contrast, revealing a gradual decrease of the apical oxygen concentration from the interface to the NNO inner layer. We observed at most six unit cells of fully reduced NNO layers in the nickelate layer stacks in the 8_4 SL sample. The residual apical oxygen atoms are visible in the first NdO_*x*_ layer at the interfaces, which is beneficial to alleviating the strong polar discontinuity at NNO/STO interfaces by providing an extra electron to compensate for the built-in electrostatic field according to the theoretical calculations.^21,28^ The observed residual apical oxygen columns toward the center of the NdO_*x*_ layer can also contribute to suppressing the polar instability. Additionally, we note that there is an asymmetrical distribution of the residual oxygen at the bottom and top interfaces, which is associated with the extent of elemental intermixing because Ti intermixing into Ni sites increases the bonding energy and hinders the removal of apical oxygen.^22^ This is in agreement with the above EELS mapping results, which shows that the proportion of cation intermixing is not homogeneous.

To explore the evolution of the electronic structure affected by the residual oxygen at the interfaces, we calculated the white-line ratios of Ni and Ti, respectively, yielding their valence variation from the STO layer to the NNO layer as displayed in Figure 3. The detailed EELS spectra of Ti-L, O-K, Ni-L, and Nd-M edges are presented in Figure S3 (SL_4_2) and Figure S4 (SL_8_4). Figures 3a and 3d present the HAADF images of SL_4_2 and SL_8_4 areas, which were used for the EELS measurements. The valence state of Ti intermixed into Ni sites in SL_4_2 tends to be 3+, while it almost maintains 4+ in SL_8_4 (Figures 3c and 3f). The valence of Ni varies between 1+ and 2+ because it depends on the extent of deintercalation of the apical oxygen in the NNO inner layer. An apparent gradual variation of the Ni valence is visible in the NNO layer for SL_8_4 as presented in Figure 3e, where the valence of Ni in the NNO inner layer tends to be 1+ and gradually increases to 2+ near the interfaces. The valence of Ni in SL_4_2 prefers to be ∼2+ in the NNO layer. The valence of Ni intermixed into the Ti sites in the STO layer is close to 3+. The reference values of the Ni-L_2,3_ white-line ratio of Ni^3+^ and Ni^2+^ are determined from a NdNiO_3_ sample^29^ and a NiO film in the Gatan EELS database,^30^ respectively. We obtain the Ni^1+^ reference from SL_8_4 because we can identify the infinite layer in the SL_8_4 sample according to the iCoM image in Figure 2h. EELS spectra of Ni-L references and the corresponding O-K edges are shown in Figure S5. According to variations of the oxygen contrast in the iCoM images and of the Ni-L_2,3_ white-line ratios, we demonstrate that the oxygen distribution and occupancy are closely related to the valence variation of Ni ions.

**Figure 3:**
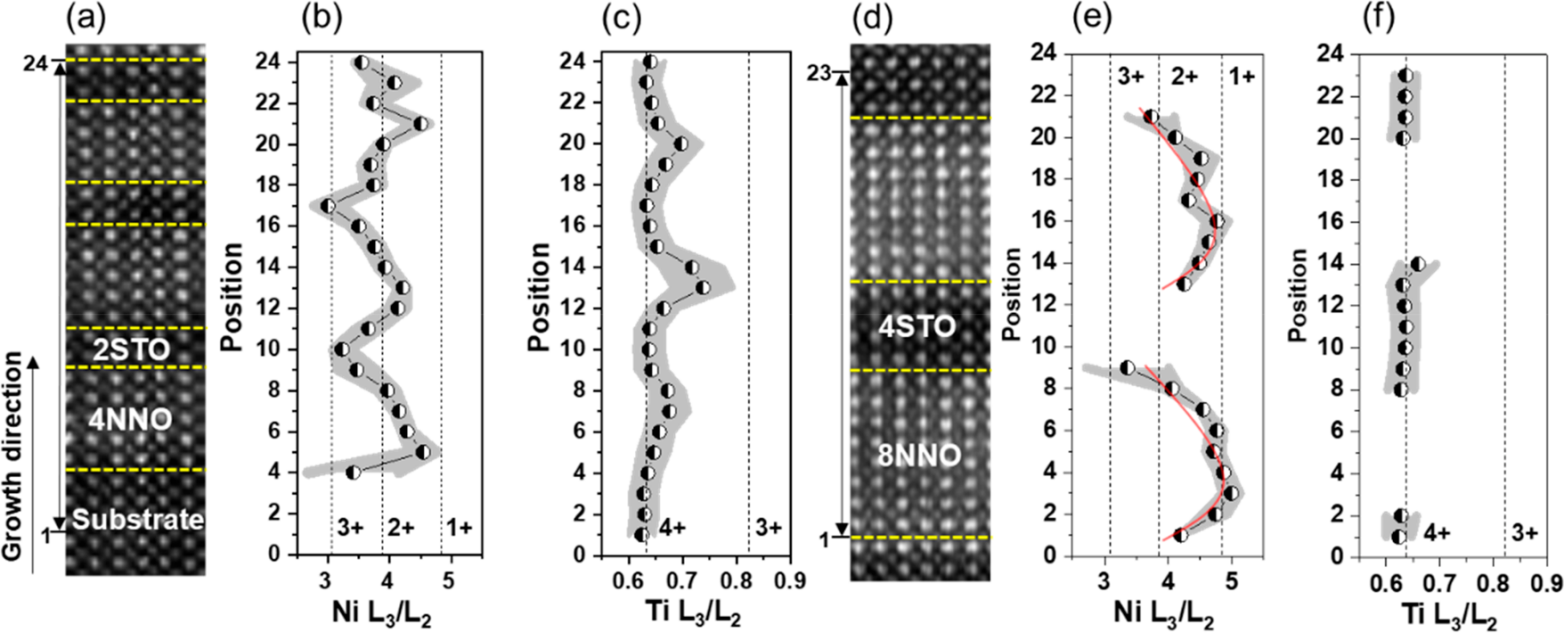
Valence variations of Ni and Ti ions across the interfaces in 4NdNiO_2_/2SrTiO_3_ (a–c) and 8NdNiO_2_/4SrTiO_3_ (d–f) superlattices. HAADF images of (a) 4NdNiO_2_/2SrTiO_3_ and (d) 8NdNiO_2_/4SrTiO_3_ superlattices. The L_3_/L_2_ white-line ratios of (b) Ni and (c) Ti in 4NdNiO_2_/2SrTiO_3_ and of (e) Ni and (f) Ti in 8NdNiO_2_/4SrTiO_3_ superlattices, respectively. The dashed reference lines for Ni^3+^, Ni^2+^, and Ni^+^ are determined from the L_3_/L_2_ white-line ratios of NdNiO_3_, NiO, and NdNiO_2_ films. The Ti^4+^ and Ti^3+^ references are from SrTiO_3_ and LaTiO_3_,^35,36^ respectively.

Furthermore, we quantify variations of cation lattice spacings in the SL_8_4 and the SL_4_2 as shown in Figure 4. Figure 4a presents a HAADF image of SL_4_2 optimized by a multiframe ADF-STEM method. Using Gaussian fitting and center-of-mass fitting based on a Python library of atomap,^31^ we calculate the in-plane and out-plane cation lattice spacings. In SL_4_2, the in-plane Nd–Nd spacing is ∼0.391 nm, which is almost the same lattice distance as of the STO substrate. In principle, the NdNiO_3_ film (lattice distance: 0.381 nm for a pseudocubic unit cell) grown on a STO (*a* = 0.3905 nm) substrate is under an epitaxial tensile strain. The in-plane lattice distance of bulk NdNiO_2_ is 0.392 nm, and the *c*-axis lattice parameter is 0.331 nm.^5^ The NdNiO_2_ film is reported to experience an epitaxial compressive strain from the STO substrate.^9^ The out-of-plane lattice spacing in a Nd_0.8_Sr_0.2_NiO_2_ film is ∼0.334 nm from XRD measurements, which has a larger *c*-axis lattice parameter than the NdNiO_2_ film due to a partial substitution of Nd by the larger Sr ion.^27^ That means a possible smaller out-of-plane lattice spacing than 0.334 nm in a NdNiO_2_ film. A smaller decrease to ∼0.376 nm of the out-plane Nd–Nd spacing occurs in SL_4_2 than the above value after the chemical reduction. The observed residual oxygen and elemental intermixing in the NNO layer can suppress the decrease of the out-of-plane lattice distance. As depicted in Figure 4b, there is a sharp reduction of the out-of-plane Nd–Nd spacing to ∼0.330 nm in SL_8_4, which is slightly smaller than the reported value from XRD measurements in a Nd_0.8_Sr_0.2_NiO_2_ film,^27^ suggesting the formation of an NNO infinite-layer structure. Additionally, a gradual decrease of the out-of-plane Nd–Nd spacing is related to the variation of oxygen occupancies from the interface to the NNO inner layer. As the interdiffusion length between STO and NNO layers is only within 2 unit cells, the variation of the out-of-plane lattice spacing is mainly ascribed to the concentration variation of the residual oxygen. Besides, the in-plane Nd–Nd distance sustains the same value as the STO substrate. Owing to the residual oxygen near the interfaces, no compressive strain may exist at the STO/NNO interfaces, while the NNO inner layers are likely under a small compressive strain. Another point is that a distinct extension of the out-of-plane lattice spacing to ∼0.4 nm occurs between the final SrO layer and the first NdO layer at partial interfaces, which is in agreement with a reported NNO single film grown on a STO substrate.^22^ This is associated with the proportion of Ni/Ti intermixing according to the DFT calculations.^22^

**Figure 4:**
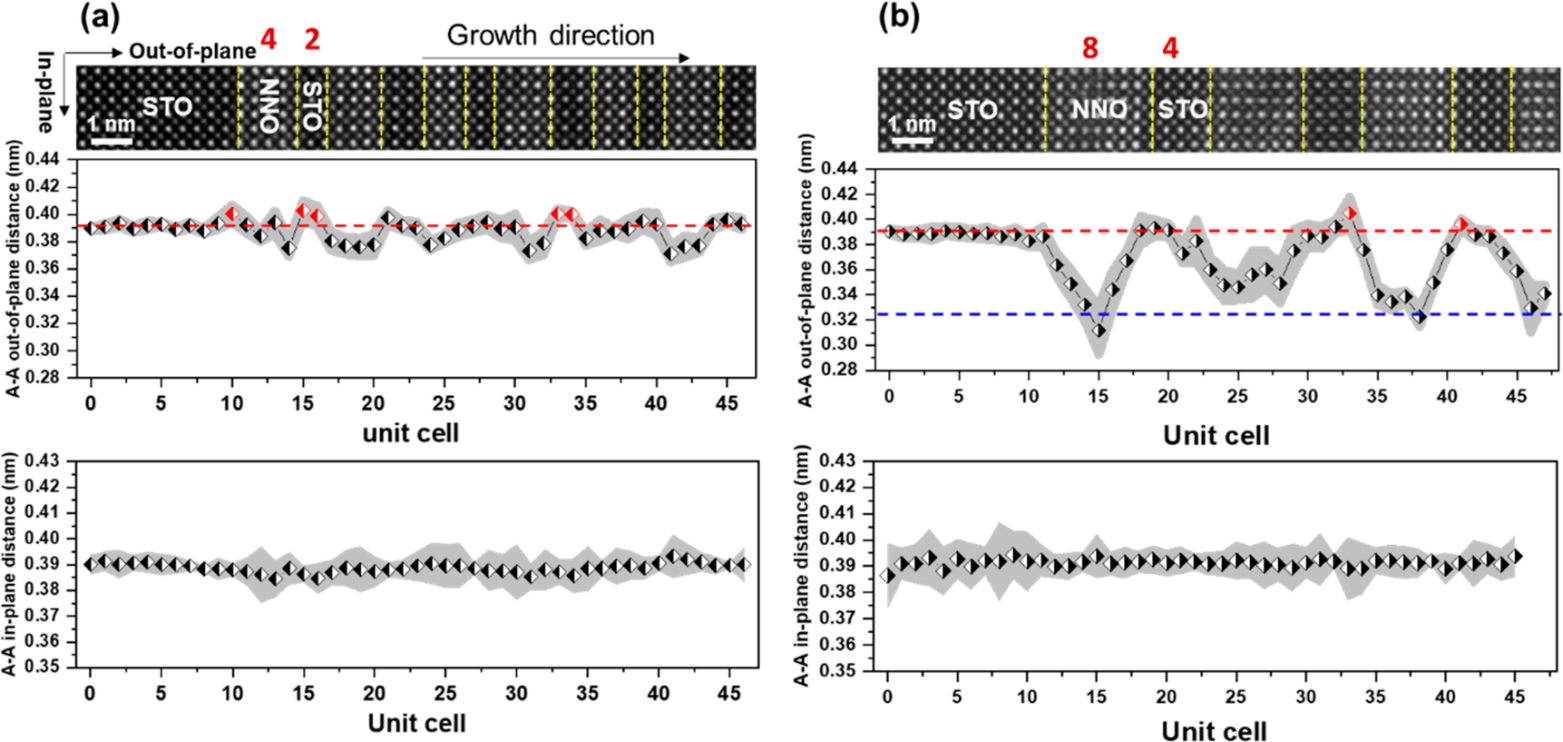
Variation of Nd–Nd lattice spacing across the NNO/STO interfaces. HAADF image of (a) 4NdNiO_2_/2SrTiO_3_ and (b) 8NdNiO_2_/4SrTiO_3_ superlattices aligned by a multiframes method and corresponding variations of in-plane and out-of-plane Nd–Nd distances, respectively. The red and blue dashed lines indicate the references of A–A out-of-plane distances from the SrTiO_3_ substrate and bulk NdNiO_2_, respectively. The red spots show larger A–A out-of-plane distances than the SrTiO_3_ substrate.

Except for the difference in the variation of lattice spacing in SL_8_4 and SL_4_2, we quantify the Ni and Ti atom displacements across the STO/NNO interfaces as shown in Figure 5. Figure 5a presents a HAADF image and a corresponding atom displacement vector map for SL_4_2. The direction and length of the red arrows indicate the direction and the distance of the atom displacements. A small displacement (below an average value of 0.01 nm) of Ni atoms points toward the NNO inner layer. In contrast, a stronger Ni displacement occurs for SL_8_4 in Figure 5b. The largest Ni displacement is ∼0.05 nm, and the average Ni displacement is ∼0.025 nm. According to the reported DFT calculation,^21^ a Ni displacement of ∼0.018 nm occurs at the NiO_2_/NdO/TiO_2_ interface owing to the residual apical oxygen at the interfaces, without considering the elemental intermixing. The value of the Ni displacement varies for different interface configurations.^21^ The displacements of Ni atoms imply the distortion of Ni–O bonds at the interfaces,^21^ indicating a stronger distortion of the Ni–O bond at the interfaces in SL_8_4 than in SL_4_2. Besides, the atomic displacements could extend to several unit cells of the NNO inner layer in SL_8_4 due to the larger interface polarity, while it is not present in SL_4_2, which would be more prounced in a NiO_2_-terminated surface layer.^14^ It is necessary to mention that the displacements of Ni atoms at the interfaces are inhomogeneous in our samples, which is affected by the inhomogeneous distribution of the residual oxygen. There is evidence of an inhomogeneous distribution of the local distortions of the oxygen sublattice at the interfaces, as shown in the enlarged iCoM images in Figure S2. Also, there is a minute displacement of the interfacial Ti in both SL_8_4 and SL_4_2. In addtition, we calculated the Ni displacement map from the HAADF image in 8NdNiO_3_/4SrTiO_3_ superlattice as shown in Figure S6, where the largest Ni shift is ∼0.025 nm, which is half of that in the reduced sample. That indirectly indicates the interface reconstruction induced by the enhanced polar discontinuity during topotactical reduction.

**Figure 5:**
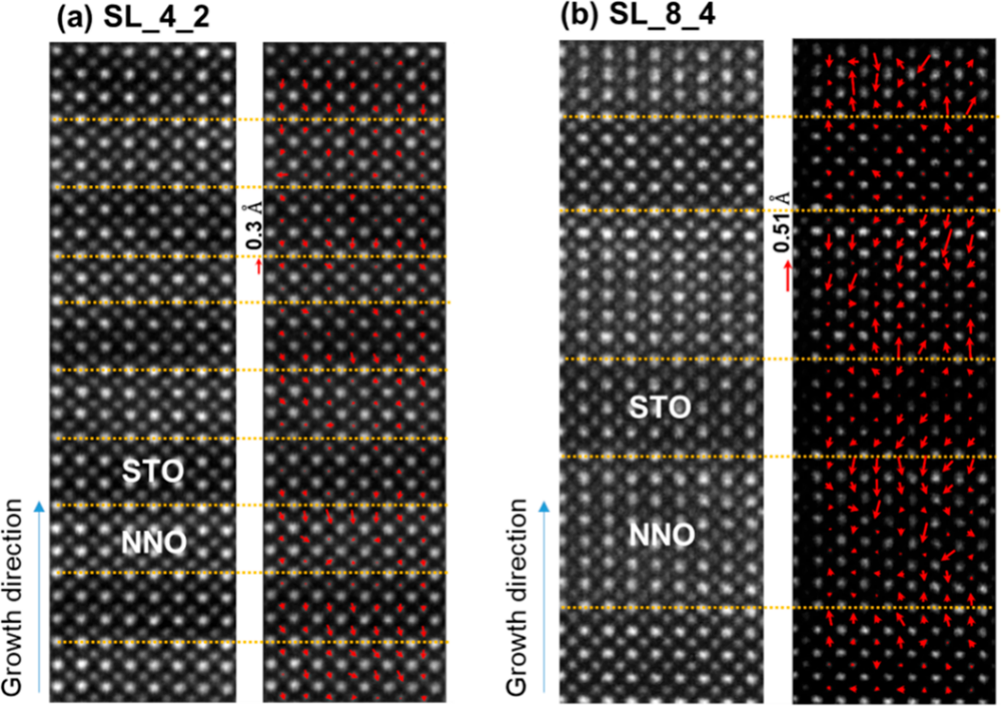
HAADF images and displacement vector maps of B atoms (B: Ni and Ti) for (a) 4NdNiO_2_/2SrTiO_3_ and (b) 8NdNiO_2_/4SrTiO_3_ superlattices.

Atomic and electronic structures are closely associated with the interface polarity, which plays a critical role in the interface properties. It can be easily affected by elemental intermixing, oxygen sublattice occupancy, and transition-metal valence variations. The interface configuration is dominated by a NiO_2_/NdO_*x*_/TiO_2_ interface in both SL_4_2 and SL_8_4 samples, where *x* is affected by the proportion of Ti intermixed into Ni sites. The EELS maps demonstrate an inhomogeneous Ni/Ti intermixing at STO/NNO interfaces. Ti intermixing into Ni sites suppresses the reduction of the apical oxygen at interfaces. The iCoM images provide direct evidence of residual oxygen at interfaces. The residual oxygen can contribute additional electrons to decrease the interface polarity by hindering the formation of a strong built-in electric field. On the other hand, the existence of residual oxygen can result in a change in the atomic structure near the interfaces. From the quantification of cation displacements, a strong Ni displacement occurs in SL_8_4. In principle, the out-of-plane Nd–Nd distance is larger in a NdNiO_3_ film than in a NdNiO_2_ film. As the chemical composition varies from {NdNiO_2_} in the NNO inner layer toward {NdNiO_3_} near the interfaces, the out-of-plane Nd–Nd distance gradually increases accordingly. Meanwhile, the asymmetric distribution of the chemical composition at each unit cell leads to a Ni displacement toward the NNO inner layer, which is in agreement with a gradual decrease of the oxygen concentration from the interface to the NNO inner layer. In contrast, the concentration variation of the residual oxygen is within two unit cells, and a relatively weaker asymmetric distribution of the chemical composition in SL_4_2 gives rise to a smaller Ni displacement. Furthermore, the atomic displacements extend to several unit cells of the NNO inner layer in the SL_8_4 sample, while it is not visible in the SL_4_2 sample due to a smaller interface polarity and the lower number of NNO layers. These differences in the atomic reconstruction at interfaces lead to a difference of the electronic structure at the interfaces. Specifically, the larger out-of-plane shift of Ni ions in the SL_8_4 sample indicates an increase of the distance between apical oxygen and Ni, increasing the orbital polarization compared with that in SL_4_2. That might enhance the electronic transport of the SL_8_4 sample.^32^ Moreover, the excess charges can be accommodated by the Ni 3d orbital due to Jahn–Teller distortions at interfaces, which is the possible reason the Ti valence tends to be 4+.^22^ In addition, we find that atomic steps were formed at the interfaces in both SL_8_4 and SL_4_2 samples as shown in Figure S7, accompanying a step distribution of the apical oxygen at the interfaces as displayed in Figure S8. That leads to a local change of the chemical composition, inhibiting the formation of a strong built-in electric field across the interfaces to a certain extent. It is worth noting that the sharp decrease of the out-of-plane lattice spacing in 8_4 SL (Figure 4) during the reduction procedure could more easily lead to lattice deformation than in 4_2 SL, especially at the interfaces with steps. As shown in the overview HAADF images in Figure S7, there is a decrease in the stability of infinite-layer phase from 4_2 SL to 8_4 SL. In addition, the microstrain from the unit cell adjacent to the step-like interface causes a local distortion of the NiO_5_ pyramid structure. Furthermore, the valence change of Ni is verified by calculating the Ni-L_2,3_ white-line ratio that is related to the electron occupancy in the Ni 3d orbital. The increase of the Ni valence at interfaces helps to screen the polar instability according to the charge transfer self-consistent model.^21,33^ The gradual variation of the valence change of Ni gradually reduces the built-in electric field between the adjacent two layers in the layer-by-layer structure. Moreover, the observed lattice expansion between the first NdO layer and the final TiO_2_ layer allows to lower the electrostatic potential energy in a point-charge lattice model.^21,34^ Thus, we demonstrated the thickness-dependent interface polarity in NNO/STO superlattices and systemically analyzed the effects of the reconstruction of the atomic and electronic structures on interface polarity.

In combination with atomic-resolution STEM-EELS and 4D-STEM, we systemically investigated the effects of oxygen distribution and occupancy, elemental intermixing, cation distortion, and layer-stack thickness on the interface polarity in NNO/STO superlattices. We directly imaged that the reduction procedure yields different local oxygen ligand field variations and a gradual variation of the oxygen content in the nickelate layers by 4D-STEM. The valence variation of Ni is closely related to the local concentration of residual oxygen. The residual oxygen, valence change of Ni, formation of atomic steps at the interfaces, and the lattice distortion contribute to the release of the polar instability at STO/NNO interfaces. Additionally, we detected a thickness-controlled interface structure and a corresponding tunable interface polarity.

## Supporting Information Available

Methods of sample synthesis, data acquirement, and data analysis; details of EELS analysis of the superlattice samples, overview HAADF images of the samples, quantitative analysis of the cation displacements in the 8NdNiO_3_/4SrTiO_3_ superlattice, and imaging of step structures at the interfaces (PDF)
