# Supplementary material for: Thickness-Dependent Interface Polarity in Infinite-Layer Nickelate Superlattices
Source: Nano Lett. 2023 Apr 7;23(8):3291–7. doi: 10.1021/acs.nanolett.3c00192 (PMC10141440; doi:10.1021/acs.nanolett.3c00192)
Supplement: Supplementary file 1 — nl3c00192_si_001.pdf [file nl3c00192_si_001.pdf]

Supporting information for

Thickness-dependent interface polarity in infinite layer nickelate superlattices

Chao Yang[1]*, Roberto A. Ortiz[1], Yi Wang[1,2], Wilfried Sigle[1], Hongguang Wang[1], Eva Benckiser[1], Bernhard Keimer[1], Peter A. van Aken[1]

[1] Max Planck Institute for Solid State Research, Stuttgart, 70569, Germany

[2] Center for Microscopy and Analysis, Nanjing University of Aeronautics and Astronautics, Nanjing, 210016, P.R. China

*Corresponding author: c.yang@fkf.mpg.de

Methods

$NdNiO_3/SrTiO_3$ superlattice samples were grown on (001)-oriented single crystal $SrTiO_3$ substrates by pulsed laser deposition (PLD) under the conditions reported in Ref.[1], followed by oxygen deintercalation through a gas-phase reaction with CaH_2 powder in a vacuum-sealed Pyrex glass tube, which was operated inside an Ar-filled glove box. The sealed Pyrex glass was heated inside a standard oven at a temperature of 280°C for a minimum of four days. To avoid differences in samples grown separately, the samples were cut into four pieces, one of which was kept pristine and the rest was chemically treated. The TEM lamellae were prepared by using a focused ion beam (FIB Scios, FEI) under high vacuum. Around 8 nm carbon was coated on the surface of the sample by a high-vacuum sputter coater (EM ACE 600, Leica) to prevent beam-induced charging effects during sample preparation. The quality of the TEM lamellae was optimized by low-energy milling and cleaning in a Fischione NanoMill® TEM specimen preparation system. The TEM measurements were performed in STEM mode using a JEOL JEM-ARM200F microscope (JEOL Co. Ltd.) equipped with a DCOR probe corrector and a Gatan GIF Quantum ERS K2 spectrometer. A condenser aperture of 30 µm was used for STEM imaging and the corresponding convergent semi-angle was 20.4 mrad. The collection semi-angle for HAADF imaging was between 83 and 205 mrad for a camera length of 2 cm. The collection semi-angle for EELS measurements was 85 mrad for a 5 mm aperture. The EELS spectra for elemental mapping were acquired at a dispersion of 0.5 eV/channel with an energy resolution of around 1 eV. The EELS spectra for computing the white-line raito were acquired at a dispersion of 0.25 eV/channel with an energy resolution of around 0.75 eV. Concerning the white-line ratio, we firstly denoise the EELS data by carefully using principal-component analysis (PCA) in the DigitalMicrograph software. Then we removed the background by using a power-law model and computed cross-sections based on the Hartree-Slater model. Afterwards, we calculated the white-line ratio from the extracted integral L_3 and L_2 white-line intensities. 4D-STEM images were acquired with a Merlin pixelated detector (256 × 256 pixels, Quantum Detectors) in 1-bit mode with continuous reading/writing at a pixel time of 48 µs. The data analysis of the 4D-STEM data sets is based on the python libraries of py4dstem[2] and fpd[3].

1. Ortiz, R. A.; Menke, H.; Misják, F.; Mantadakis, D. T.; Fürsich, K.; Schierle, E.; Logvenov, G.; Kaiser, U.; Keimer, B.; Hansmann, P.; Benckiser, E., Superlattice approach to doping infinite-layer nickelates. *Phys. Rev. B* **2021,** *104* (16), 165137.
2. Savitzky, B. H.; Zeltmann, S. E.; Hughes, L. A.; Brown, H. G.; Zhao, S.; Pelz, P. M.; Pekin, T. C.; Barnard, E. S.; Donohue, J.; Rangel DaCosta, L.; Kennedy, E.; Xie, Y.; Janish, M. T.; Schneider, M. M.; Herring, P.; Gopal, C.; Anapolsky, A.; Dhall, R.; Bustillo, K. C.; Ercius, P.; Scott, M. C.; Ciston, J.; Minor, A. M.; Ophus, C., py4DSTEM: A Software Package for Four-Dimensional Scanning Transmission Electron Microscopy Data Analysis. *Microsc. Microanal.* **2021,** *27* (4), 712-743.
3. Paterson, G. W.; Webster, R. W. H.; Ross, A.; Paton, K. A.; Macgregor, T. A.; McGrouther, D.; MacLaren, I.; Nord, M., Fast Pixelated Detectors in Scanning Transmission Electron Microscopy. Part II: Post-Acquisition Data Processing, Visualization, and Structural Characterization. *Microsc. Microanal.* **2020,** *26* (5), 944-963. 1.

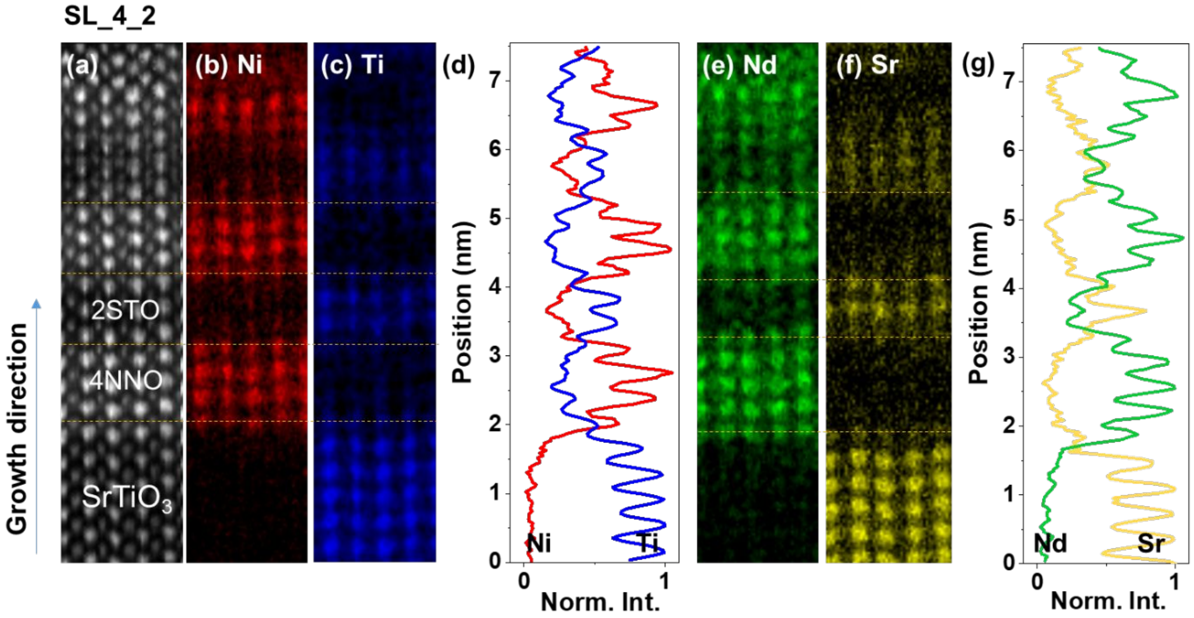

Figure S1. Elemental distribution across the interfaces of a $4NdNiO_2/2SrTiO_3$ superlattice. (a) An HAADF image for the acquisition of the EELS mapping. EELS maps of (b) Ni (red), (c) Ti (blue), (e) Nd (green), and (f) Sr (yellow). Normalized intensity line profiles of (d) Ni and Ti, and (g) Nd and Sr.

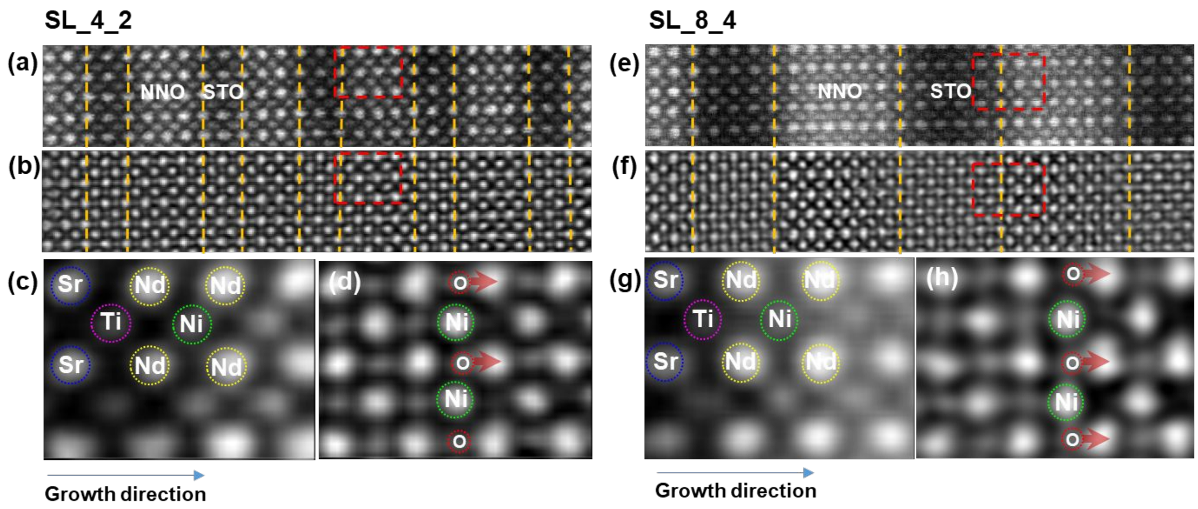

Figure S2. Oxygen octahedra distortion at the interfaces in $4NdNiO_2/2SrTiO_3$ and $8NdNiO_2/4SrTiO_3$ superlattices. (a) ADF image and (b) iCoM image reconstructed from a 4D-STEM dataset of a $4NdNiO_2/2SrTiO_3$ superlattice. The enlarged (c) ADF image and (d) iCoM image are from the region marked with red dashed boxes in (a) and (b), respectively. (e) ADF image and (f) iCoM image reconstructed from a 4D-STEM dataset of an $8NdNiO_2/4SrTiO_3$ superlattice. The enlarged (g) ADF image and (h) iCoM image are from the region marked with red dashed boxes in (e) and (f), respectively.

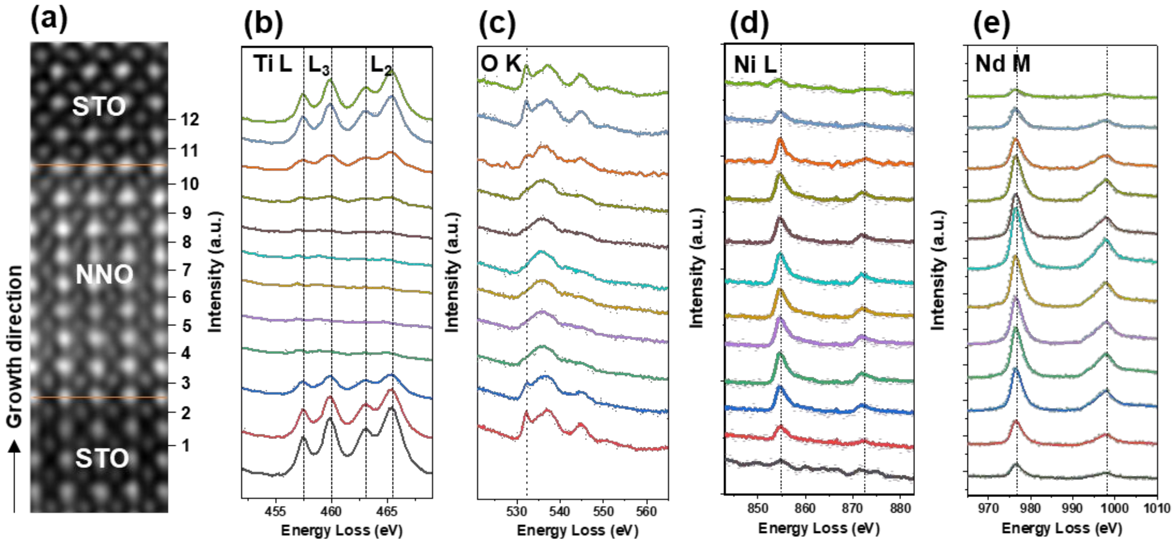

Figure S3. (a) An ADF image for EELS data analyses of the 8_4 SL sample. Corresponding EELS spectra of the (b) Ti-$L_{2,3}$ edges, (c) O-K edges, (d) Ni-$L_{2,3}$ edges, and (e) Nd-$M_{4,5}$ edges.

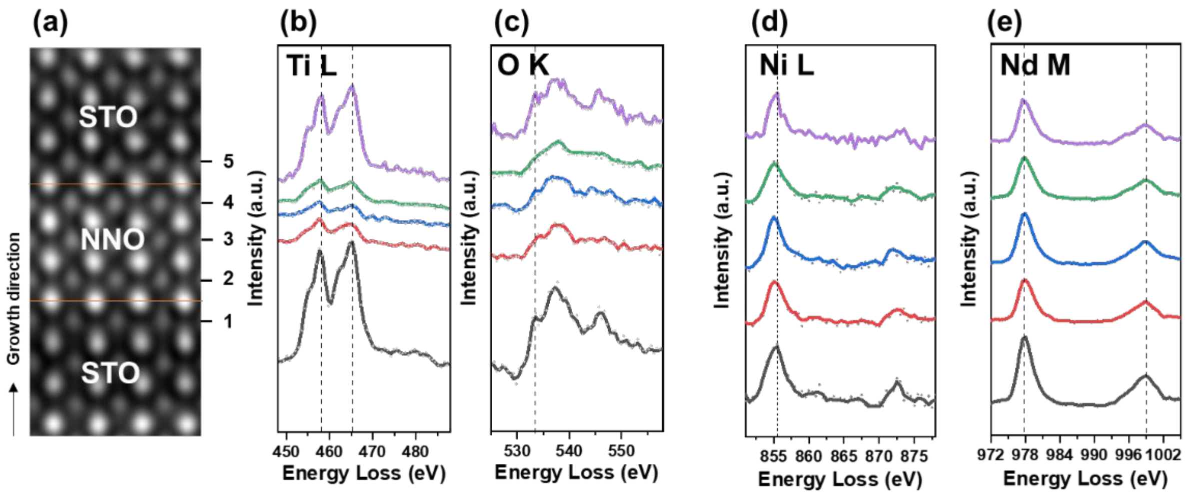

Figure S4. (a) An ADF image for EELS data analyses of the 4_2 SL sample. Corresponding EELS spectra for the (b) Ti-$L_{2,3}$ edges, (c) O-K edges, (d) Ni-$L_{2,3}$ edges, and (e) Nd-$M_{4,5}$ edges.

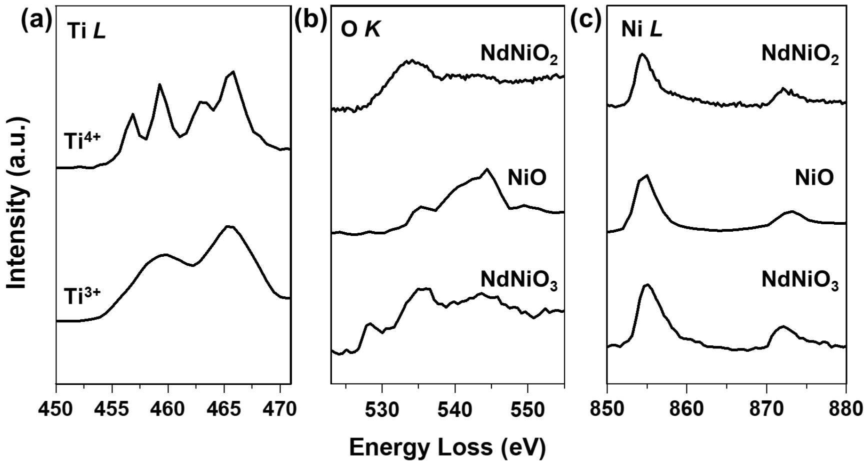

Figure S5. The references of the EELS spectra for (a) Ti-$L_{2,3}$, (b) O-K, and (c) Ni-$L_{2,3}$ edges.

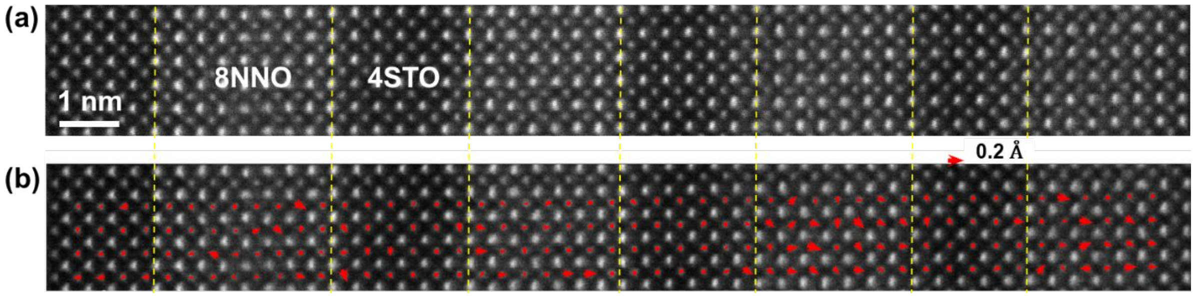

Figure S6. HAADF image (a) and displacement vector map (b) of B atoms (B: Ni, and Ti) for the $8NdNiO_3/4SrTiO_3$ superlattice.

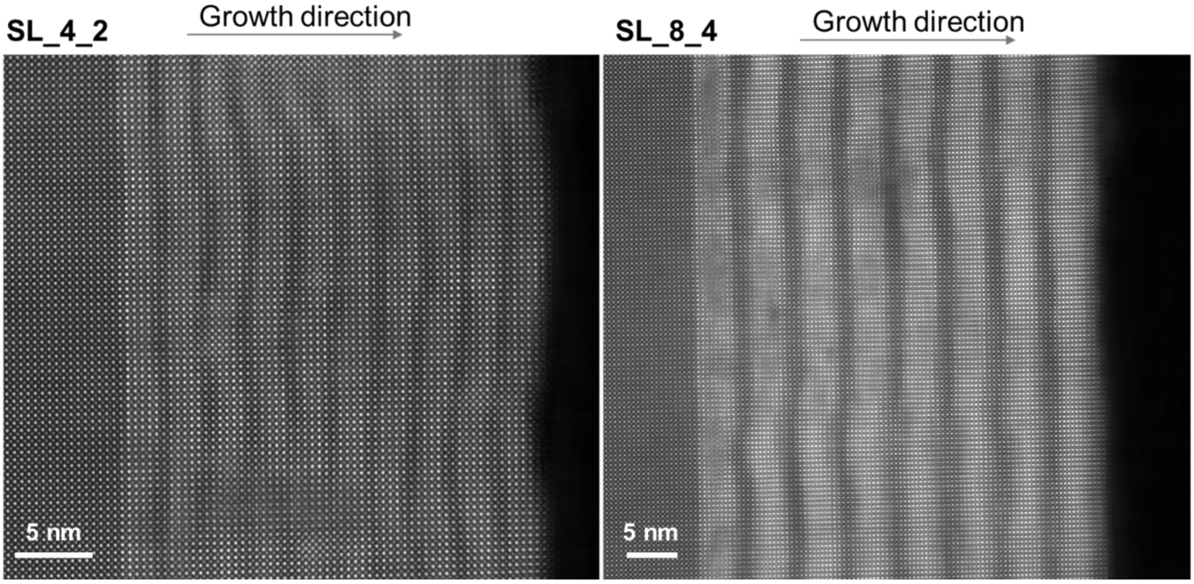

Figure S7. Overview HAADF images of $4NdNiO_2/2SrTiO_3$ and $8NdNiO_2/4SrTiO_3$ superlattices.

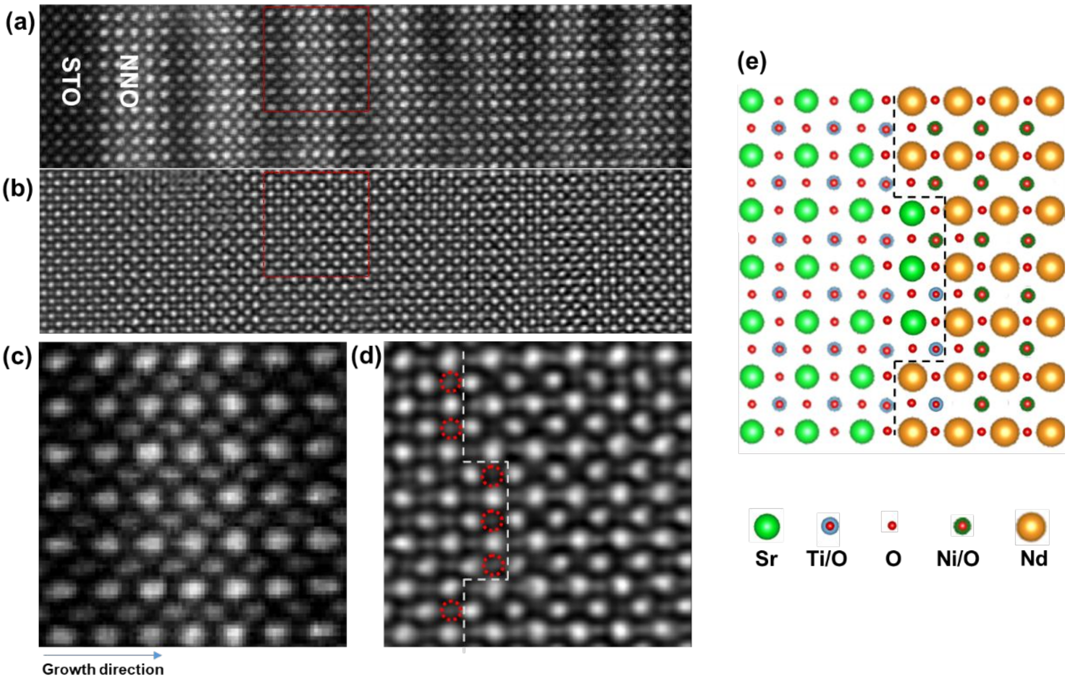

Figure S8. (a) ADF image and (b) iCoM image reconstructed from a 4D-STEM dataset of a $4NdNiO_2/2SrTiO_3$ superlattice. (c) The enlarged ADF image and (d) iCoM image are from the regions marked with red dashed boxes in (a) and (b), respectively. The red circles indicate the presence of apical oxygen atoms. (e) A schematic diagram of the interface structure with steps.
